# Supplementary material for: SOFA Score Plus Impedance Ratio Predicts Mortality in Critically Ill Patients Admitted to the Emergency Department: Retrospective Observational Study
Source: Healthcare (Basel). 2022 Apr 27;10(5):810. doi: 10.3390/healthcare10050810 (PMC9140899; doi:10.3390/healthcare10050810)
Supplement: Supplementary file 1 [file healthcare-10-00810-s001.zip › healthcare-1673018-supplementary.pdf]

SUPPLEMENTARY DATA

Supplementary Table S1. Comparison bioimpedance variables at admission to ED in survivors and non-survivors.

	30-days Mortality			In-Hospital Mortality			In-ED Mortality		
	**No n=165**	**Yes n=75**	***p* value**	**No n=181**	**Yes n=59**	***p* value**	**No n=216**	**Yes n=24**	***p* value**
Z to 5 kHz, Ω	604 (512.5-724.5)	557 (462-651)	0.031	596 (494-711)	559 (478-677)	0.211	596 (499-704)	495.5 (378-683)	0.050
Z to 50 kHz, Ω	545 (467.5-654)	520 (432-599)	0.079	541 (455.5-650.5)	520 (432-626)	0.313	543 (466-649)	446 (368-614)	0.031
Z to 100 kHz, Ω	512 (447-626.5)	510 (417-581)	0.202	511 (442-625.5)	511 (418-609)	0.500	513 (442-621)	430.5 (392-597)	0.036
Z to 200 kHz, Ω	489 (425.5-604.5)	493 (399-562)	0.340	488 (419.5-600)	496 (399-588)	0.641	494 (423-595)	412.5 (343-576)	0.052
Resistance	539 (464.3-650)	517 (429-596)	0.070	536.4 (536.4-647.1)	517 (429.7-619.2)	0.297	538.8 (459-644)	442.6 (361-607)	0.021
Reactance	49.5 (29.9-64.7)	28.8 (19.8-48.5)	<0.001	46.7 (28.7-63.4)	31.9 (19.8-48.5)	<0.001	43.8 (27.862.1)	23.8 (16.3-38.4)	0.001
Phase angle	4.7 (3.6-6.1)	3.3 (2.5-4.9)	<0.001	4.5 (3.4-6)	3.2 (2.5-5.1)	<0.001	4.4 (3.2-5.9)	3.2 (2.2-4.3)	0.001

Data are expressed by median and IQR (1st-3er quartile); ED: Emergency department, , Z: impedance.
